# Learning Architecture for Brain Tumor Classification Based on Deep Convolutional Neural Network: Classic and ResNet50

**DOI:** 10.3390/diagnostics15050624

**Published:** 2025-03-05

**Authors:** Rabei Raad Ali, Noorayisahbe Mohd Yaacob, Marwan Harb Alqaryouti, Ala Eddin Sadeq, Mohamed Doheir, Musab Iqtait, Eko Hari Rachmawanto, Christy Atika Sari, Siti Salwani Yaacob

**Affiliations:** 1 Technical Engineering College for Computer and AI, Northern Technical University, Mosul 41000, Iraq; rabei@ntu.edu.iq; 2 Center for Software Technology and Management (SOFTAM), Faculty of Information Science and Technology, University Kebangsaan Malaysia (UKM), Selangor 43600, Malaysia; 3 Department of English Language-Literature and Translation, Zarqa University, Zarqa 13110, Jordan; mqaryouti@zu.edu.jo (M.H.A.); alaeddin71@yahoo.com (A.E.S.); 4 Department of Technology Management, Universiti Teknikal Malaysia Melaka, Malacca 76100, Malaysia; 5 Department of Data Science and Artificial Intelligence, Zarqa University, Zarqa 13110, Jordan; migtait@zu.edu.jo; 6 Faculty of Computer Science, Universitas Dian Nuswantoro, Semarang 50131, Indonesia; eko.hari@dsn.dinus.ac.id (E.H.R.); atika.sari@dsn.dinus.ac.id (C.A.S.); 7 Department of Computer Science, Faculty of Computing, Universiti Malaysia Pahang Al-Sultan Abdullah, Pahang 26600, Malaysia; sitisalwani@umpsa.edu.my

**Keywords:** deep learning, Convolutional Neural Networks, ResNet-50, image classification, magnetic resonance imaging

## Abstract

**Background:** Accurate classification of brain tumors in medical images is vital for effective diagnosis and treatment planning, which improves the patient’s survival rate. In this paper, we investigate the application of Convolutional Neural Networks (CNN) as a powerful tool for enhancing diagnostic accuracy using a Magnetic Resonance Imaging (MRI) dataset. **Method:** This study investigates the application of CNNs for brain tumor classification using a dataset of Magnetic Resonance Imaging (MRI) with a resolution of 200 × 200 × 1. The dataset is pre-processed and categorized into three types of tumors: Glioma, Meningioma, and Pituitary. The CNN models, including the Classic layer architecture and the ResNet50 architecture, are trained and evaluated using an 80:20 training-testing split. **Results:** The results reveal that both architectures accurately classify brain tumors. Classic layer architecture achieves an accuracy of 94.55%, while the ResNet50 architecture surpasses it with an accuracy of 99.88%. Compared to previous studies and 99.34%, our approach offers higher precision and reliability, demonstrating the effectiveness of ResNet50 in capturing complex features. **Conclusions:** The study concludes that CNNs, particularly the ResNet50 architecture, exhibit effectiveness in classifying brain tumors and hold significant potential in aiding medical professionals in accurate diagnosis and treatment planning. These advancements aim to further enhance the performance and practicality of CNN-based brain tumor classification systems, ultimately benefiting healthcare professionals and patients. For future research, exploring transfer learning techniques could be beneficial. By leveraging pre-trained models on large-scale datasets, researchers can utilize knowledge from other domains to improve brain tumor classification tasks, particularly in scenarios with limited annotated data.

## 1. Introduction

A brain tumor is an abnormal growth of cells within the brain that can disrupt its normal functioning [1]. Brain tumors can be classified into benign (non-cancerous) and malignant (cancerous) tumors [2]. Malignant tumors can invade surrounding tissues and form secondary tumors (metastasis). The incidence of brain tumor cases worldwide has shown a significant increase in recent years [3]. According to the latest data, millions of new cases of brain tumors are diagnosed each year across the globe [3,4]. Several risk factors associated with the development of brain tumors include genetic factors, exposure to radiation, family history, and unhealthy lifestyle choices [5]. Despite ongoing research and advancements in treatment, brain tumors remain a complex and challenging disease to treat. Diagnosis and monitoring of brain tumors have greatly benefited from advancements in medical technology, particularly Magnetic Resonance Imaging (MRI) [6,7]. MRI utilizes powerful magnets and radio waves to generate detailed images of the internal structures of the brain [8].

MRI plays a pivotal role in characterizing brain tumors, providing high-resolution images that enable healthcare professionals to accurately determine the tumor’s location, size, and extent [9]. Moreover, MRI scans assist in distinguishing between benign and malignant tumors, a critical factor in determining the appropriate treatment strategy. Additionally, MRI is employed in the surgical planning process, aiding neurosurgeons in precisely targeting the tumor and minimizing damage to healthy brain tissue. Furthermore, MRI is invaluable in post-treatment follow-ups as it enables physicians to assess treatment effectiveness and monitor any potential tumor recurrence [10]. In conclusion, the advancements in MRI technology have revolutionized the diagnosis, treatment planning, and monitoring of brain tumors, ultimately resulting in enhanced patient outcomes [11].

Convolutional Neural Networks (CNN) have proven to be highly beneficial in the realm of medical imaging analysis, specifically in the detection and classification of brain tumors [12]. CNNs, which are a type of deep learning algorithm, enable medical professionals to harness the power of machine learning to aid in the interpretation and diagnosis of brain tumor cases [13]. By training CNNs on extensive datasets of MRI images, these algorithms can acquire knowledge of intricate patterns and features associated with various types of brain tumors. Consequently, CNNs can automatically analyze new MRI scans, thereby providing valuable insights about tumor characteristics such as size, location, and rate of growth. The integration of CNN technology with MRI scans effectively enhances the efficiency and accuracy of brain tumor diagnosis, thus empowering healthcare providers to make well-informed decisions and devise tailored treatment plans [14]. Moreover, ongoing research is dedicated to the development of CNN models capable of predicting treatment outcomes and facilitating the advancement of targeted therapeutic approaches. In summary, the fusion of CNN technology with MRI scans holds tremendous potential for advancing the field of brain tumor diagnosis and treatment, ultimately resulting in enhanced patient care and improved outcomes [15].

The paper is structured into different sections, each serving a specific purpose. In Section 2, the authors provide a Literature review, offering an overview of the relevant background and existing knowledge in the field. Section 3 introduces the proposed method, detailing the CNN application for brain tumor classification using the pre-processing, Extraction Features, Confusion Matrix, and dataset of MRI images. Section 4 describes the experimental setup, including the training and testing procedures, and the data split used for evaluation. Which includes the results and analysis of the experiments. The accuracies of both the Classic layer architecture and the ResNet50 architecture are reported, along with other performance metrics like precision, recall, specificity, and F1-score, providing a comprehensive evaluation of the CNN models’ effectiveness in classifying brain tumors. The final Section presents the conclusion, summarizing the key findings and highlighting the significance of the study.

## 2. Related Work

Kesav et al. [16], the authors proposed CNN architecture that achieved state-of-the-art results in image recognition. Similarly, Toptas et al. [17] utilized CNNs to address the problem of object detection, demonstrating significant improvements in accuracy compared to traditional methods. Furthermore, CNNs have also been widely used in medical imaging analysis, including the detection and classification of tumors in MRI scans. These studies highlight the effectiveness and versatility of CNNs in handling diverse visual recognition tasks, motivating further exploration and advancements in the field.

CNN has been extensively studied and widely applied in various fields of computer vision and pattern recognition [18]. Numerous works have focused on enhancing the capabilities and performance of CNNs. Rahman et al. [19], researchers have explored different architectures and network designs to improve feature extraction and representation learning. Vankdothu et al. [20], the researcher’s interest is the integration of CNNs with other deep learning models, such as recurrent neural networks (RNNs), to tackle sequential or temporal data analysis tasks. Furthermore, the development of transfer learning techniques, where pre-trained CNN models are utilized as a starting point for new tasks, has proven beneficial for addressing data scarcity and accelerating model convergence [21]. These related works highlight the ongoing efforts in advancing CNNs and their applications, contributing to the continuous improvement and innovation in the field of deep learning. To compare the novelty, the author selected several researchers who employed the same type of dataset but with different methods.

Lakshmi and Rao [22], the implementation of Deep CNN with the GoogleNet Inception v3 architecture on a dataset consisting of 3064 MRI images yielded an impressive accuracy rate of 89%. The utilization of the sophisticated Inception v3 model allowed effective feature extraction and representation, enabling the network to discern intricate patterns and variations within the MRI data. In the realm of medical image analysis, various researchers have explored different methods to achieve accurate results.

Ranjbarzadeh et al. [23], employed the Cascade CNN technique on the Brats 2018 dataset, attaining a remarkable accuracy of 92.03%. These findings exemplify the versatility of deep learning approaches in medical imaging, where different methodologies can lead to substantial variations in accuracy. It underscores the significance of method selection and dataset choice in the pursuit of reliable and precise medical image analysis systems. As research in this field continues to evolve, these benchmark results serve as crucial reference points for future investigations aiming to enhance the diagnostic capabilities of medical imaging technologies.

## 3. Proposed Method

### 3.1. Datasets

**Figure 1:**
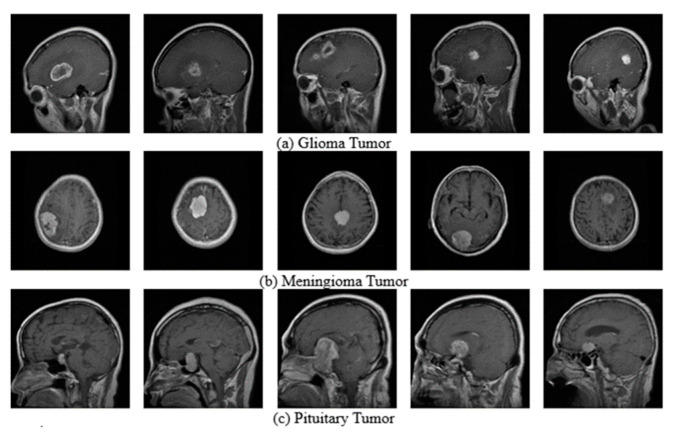
Sample of Brain Tumor Image.

The utilization of the Brain tumor dataset obtained from Kaggle [24,25], which comprises MRI images with a resolution of 512 × 512 × 1, is a valuable resource for studying and analyzing brain tumors. The dataset consists of diverse cases involving three types of tumors: Glioma, Meningioma, and Pituitary tumors as shown in Figure 1.

To facilitate the analysis process, the images are resized and standardized to a resolution of 200 × 200 × 1. This preprocessing step ensures consistency in the dataset by reducing the image size while preserving the essential features and characteristics of the tumors. The availability of this dataset allows researchers, medical professionals, and machine learning practitioners to explore various image processing techniques, develop accurate tumor detection and classification algorithms, and contribute to advancements in the field of brain tumor diagnosis and treatment. Sample datasets can be seen above.

### 3.2. Pre-Processing

Pre-processing is a crucial stage in image processing aimed at preparing images before further analysis or processing takes place [26]. Typically, preprocessing involves a series of steps such as contrast adjustment, resizing, thresholding, segmentation, and image enhancement [26,27,28,29,30]. These steps are designed to eliminate noise, enhance important features, improve the contrast between objects and the background, and facilitate object identification and analysis within the image. Preprocessing may also involve mathematical and statistical operations, such as dilation and erosion, feature extraction, normalization, and color space transformation [17]. Based on the proposed method, the following mathematical expression algorithms are utilized to carry out the preprocessing steps:

1. Input Image: W_in×H_in×C_in=200×200×1.
2. Image Thresholding with a specified threshold using Black and White: bw$\left(i,j\right):$ if input image$\left(i,j\right)$ > *threshold*, then 1; if Input Image$\left(i,j\right)\leq$ *Threshold*, then 0; else “Wrong Input”
3. Labeling Connected Objects: *label*$\left(i,j\right):$ if bw$\left(i,j\right)$ is part of a connected object labeled as k than k; if bw$\left(i,j\right)$ is part of the background than 0;
4. Computing the area and convexArea property from the label:
  (1) $$CA\left(k\right):\sum_{\left(i,\;j\right)}\left(label\left(i,j\right)=k\right)\times pixelArea\;\mathrm{Get}\;\mathrm{the}\;\mathrm{convexArea}$$
  (2) $$area\left(k\right):\;\sum_{\left(i,\;j\right)}\left(label\left(i,\;j\right)=k\right)\;\mathrm{Get}\;\mathrm{Area}$$
5. Computing the Solidity property from the label:
  (3) $$density\left(k\right)=\frac{area(k)}{CA(k)}\leftarrow \;\mathrm{Get}\;\mathrm{density}$$
6. Creating a binary vector based on the Solidity property of density: high_dense_area(k): if *density*(k) > Threshold, then 1; if *density*(k)≤*threshold*, then 0.
7. Finding the maximum area value among the objects with high density: MA=area(k)|high_dense_area(k)=1.
8. Selecting the label(s) with the maximum area: tumor_label=k|area(k)=max_area.
9. Creating a binary image with the selected label(s) after dilation: Tumor detected $\left(i,j\right):$= if label (i,j) is an element of tumor_label after dilation then 1, if label (i,j) is not an element of tumor_label after dilation then 0.

Where, i and j are pixel indices are in the image. The threshold is the threshold value used in the thresholding step. k represents the label of a connected object. convexArea (k) denotes the area of the region that encloses the object labeled as k after convex hull operation. Tumor detected (i,j) represents the binary image obtained after segmenting the tumor, where the value is 1 for pixels belonging to the tumor object and 0 for pixels that are not part of the tumor object.

### 3.3. Confusion Matrix

Confusion matrix is a tabular representation used to evaluate the performance of a classification model [31]. It summarizes the predicted and actual class labels for a given dataset. The matrix is organized into for quadrants: true positive (TP), true negative (TN), false positive (FP), and false negative (FN) represents the number of instances correctly predicted as positive, (*TN*) represents the number of cases correctly predicted as negative, FP represents the number of instances incorrectly predicted as positive, and (*FN*) represents the number of instances incorrectly predicted as negative. The confusion matrix allows for a comprehensive assessment of the model’s performance, including accuracy, precision, recall, and F1 score. Analyzing the values in the confusion matrix makes it possible to gain insights into the model’s strengths and weaknesses in correctly classifying the different classes. Equation of Evaluation can be seen below.

(4) $$Accuracy=\frac{\left(TP+TN\right)}{\left(TP+TN+FP+FN\right)}$$

(5) $$Precision=\frac{TP}{\left(TP+FP\right)}$$

(6) $$Recall=\frac{TP}{\left(TP+FN\right)}$$

(7) $$F1-score=\frac{2\;*\;(Precision\;*\;Recall)\;}{\left(Precision+Recall\right)}$$

### 3.4. Convolutional Neural Network (CNN)

CNN are a formidable class of deep learning models that find widespread application in computer vision tasks [32]. CNNs are specifically designed to autonomously learn and extract meaningful features from input images. They consist of multiple layers, including convolutional layers, pooling layers, and fully connected layers. In a CNN, the convolutional layers apply a set of adaptable filters to the input image, extracting local patterns and features. These filters discern various visual patterns, such as edges, corners, and textures. The pooling layers subsequently down sample the spatial dimensions of the feature maps, thereby reducing computational complexity and capturing the most salient information. Finally, the fully connected layers, located at the end of the network, amalgamate the extracted features and execute the ultimate classification or regression task. By leveraging shared weights and local receptive fields, CNN efficiently acquire knowledge about intricate patterns and invariant representations, rendering them highly suitable for tasks such as image classification, object detection, and image segmentation [33]. Training at CNN entails optimizing the network parameters through backpropagation and gradient descent, enabling the network to minimize the disparity between its predictions and the true labels in the training data. Additionally, CNNs can derive benefits from techniques such as data augmentation, regularization, and transfer learning, thereby enhancing their performance and generalization capabilities. In conclusion, CNNs have brought about a revolutionary transformation in the field of computer vision, yielding state-of-the-art results in diverse visual recognition tasks. Their innate ability to autonomously learn and extract meaningful features from images has significantly advanced the capacities of computer systems in comprehending and interpreting visual information.

(8) $$\left(f\times w\right)\left[i,j\right]=\sum_{m,n}f\left[m,n\right]\times w\left[i-m,\;j-n\right]$$

In this study, the Convolution Layers utilized can be observed as follows. The output value at the position (i,j) after applying convolution to the input data f using the convolutional kernel w is represented as (f×w)[i,j]. The convolution operation involves summing the products of the input data and the corresponding elements of the kernel over the spatial dimensions. This process applies the kernel to the relevant receptive field for each output position.

### 3.5. Convolution Layers

**Figure 2:**
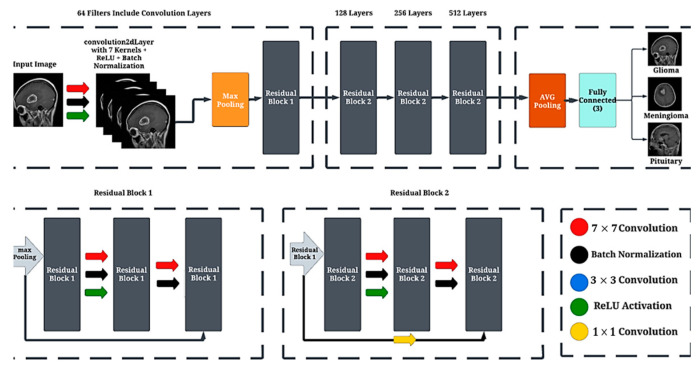
CNN with ResNet-50 Architecture.

The convolutional layer in Figure 2 describes architecture plays a crucial role in extracting relevant features from the input data. With a kernel size of 7 and 64 filters, this layer performs convolution by sliding the kernel over the input image. Each filter applies a set of weighted connections to a local receptive field, capturing specific patterns and features. The stride of 2 and padding of 3 control the spatial down sampling and spatial dimensions of the output feature maps. By convolving the input data with adaptable filters, the convolutional layer effectively learns and represents visual patterns, enabling subsequent layers to make informed decisions based on these extracted features. The combination of convolution, batch normalization, and ReLU activation in this layer aids in enhancing the network’s ability to discern and capture relevant information from the input data.

### 3.6. Residual Block Layer

A residual block, which is a fundamental component of the CNN architecture described in Figure 2, is designed to address the issue of vanishing gradients and enable the network to learn more effectively. It consists of several sequential layers, including convolutional layers, batch normalization layers, and activation layers. The input data is processed through these layers, and the output is obtained by adding the input data to the output of the final convolutional layer, using a skip connection. This skip connection allows the network to learn residual mappings, capturing the residual information that needs to be added to the original input for better feature representation. By incorporating residual blocks into the CNN architecture, the network can effectively capture and propagate relevant information, enabling more efficient training and improved overall performance in tasks such as image classification and object recognition [34].

### 3.7. Batch Normalizatio

Batch normalization is a crucial layer in the CNN architecture described in Figure 2. It operates by normalizing the activations of the previous layer within a mini-batch of training examples. This normalization step helps in reducing the internal covariate shift, which is the phenomenon of the distribution of layer activations changing during training.

ReLU activation function is applied in the CNN architecture to introduce non-linearity and enhance the network’s learning capability. ReLU is a simple yet powerful activation function that operates elementwise on the output of each convolutional layer and sets negative values to zero while preserving positive values. This activation function helps to overcome the vanishing gradient problem, allowing for efficient gradient propagation and faster convergence during training.

### 3.8. Max Pooling

Max pooling is a crucial operation in the CNN architecture described in Figure 2. It is performed using the maxPooling2dLayer, which reduces the spatial dimensions of the feature maps while preserving the most prominent features. During the max pooling process, a rectangular window of a specified size, typically 3 × 3, slides over the input feature map. The maximum value within the window is selected and retained at each position, while the other values are discarded. This down-sampling operation helps to reduce the computational complexity of the network and enhances its robustness against small spatial variations in the input data

### 3.9. Fully Connected Layer

Fully connected layer in Figure 2. Architecture is crucial for integrating the extracted features and making final predictions. It connects every neuron from the previous layer to each neuron in the following layer. By doing so, it enables the network to learn complex relationships and patterns within the extracted features. The fully connected layer performs a weighted sum of the inputs and applies an activation function to generate the output.

## 4. Results and Discussion

In this study, the performance and efficacy of both the Classic architecture and the ResNet50 architecture were thoroughly investigated and compared in the context of brain tumor classification. The experiments were conducted using a dataset consisting of 4922 brain tumor images, which were divided into 80% training data and 20% testing data. The results obtained from the evaluation process demonstrated that the ResNet50 architecture outperformed Classic architecture in terms of accuracy, precision, recall, and F1 score. The ResNet50 model exhibited superior capability in capturing intricate and abstract features from the tumor images, resulting in higher classification accuracy. Table 1 provides an overview of the hyperparameters utilized for the CNN models discussed earlier, which were selected as the training options for the experiments. The table presents a comprehensive list of these hyperparameters, which include parameters such as the learning rate, batch size, number of epochs, and optimizer used for training the CNN models.

**Figure 3:**
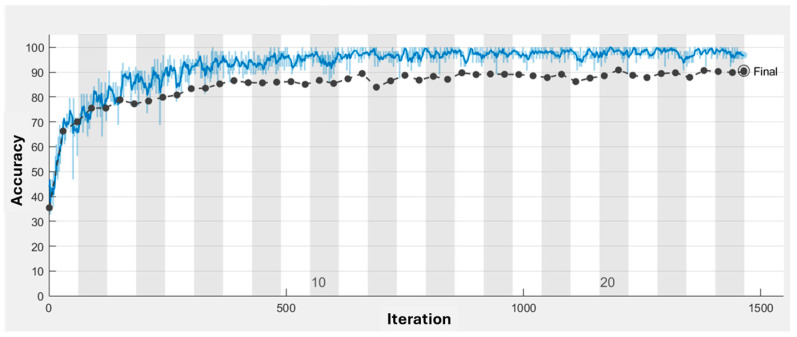
Accuracy Training Progress with Resnet-50 Architecture.

**Figure 4:**
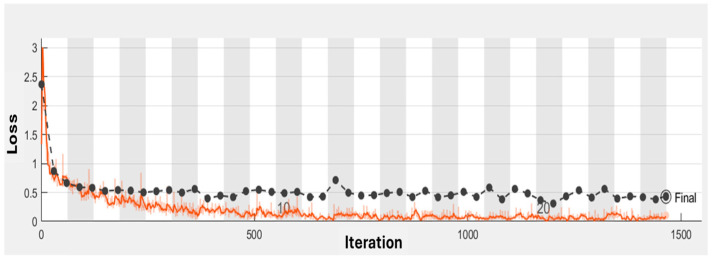
Loss Training Progress with Resnet-50 Architecture.

In addition to the parameters described in Table 1, the ex-per mentation with deep learning CNN models also incorporated the implementation of the ResNet-50 layer. In our experiments, the input images were resized to a resolution of 200 × 200 × 1 to ensure consistency across both models, Classic CNN and ResNet50. The resizing process preserved the essential features of the tumors by maintaining the aspect ratio and padding the images where necessary. This was followed by normalization, where pixel values were scaled between 0 and 1 by dividing by 255, ensuring that the images were suitable for the neural networks. The Classic CNN architecture consists of several convolutional layers (e.g., 3 × 3 × 8, 3 × 3 × 16, 3 × 3 × 32) followed by batch normalization, ReLU activation, and max pooling layers, culminating in a fully connected layer and softmax classification. For ResNet50, which uses residual blocks, the architecture includes convolutional layers, batch normalization, ReLU activations, and average pooling, with several residual blocks at different depths (e.g., 64 × 64, 128 × 128, 256 × 256). Despite the image resizing, we verified that the key features, such as tumor shapes and boundaries, remained identifiable in the processed images. Additionally, the model’s performance did not significantly degrade, confirming that the image preprocessing steps, including resizing, were effective in preserving the essential tumor characteristics. The ResNet-50 layer is a widely acclaimed convolutional neural network architecture renowned for its proficiency in addressing complex image classification tasks. By incorporating ResNet-50, the CNN model benefited from increased depth and a structured flow of information. This inclusion of the ResNet-50 layer proved to be a noteworthy enhancement, enabling the model to effectively tackle challenging learning tasks and achieve more accurate results. Based on Figure 3 and Figure 4, the training progress mentioned above, the evaluation of the models revealed significant accurate results. Classic layer architecture achieved an accuracy of 94.55%, while the implementation of the ResNet50 architecture resulted in an improved accuracy of 99.88%. These findings indicate the superiority of the Res-Net50 architecture in accurately classifying brain tumors compared to the Classic layer architecture. The higher accuracy obtained with ResNet50 demonstrates its effectiveness in capturing intricate patterns and features in the dataset, leading to more precise tumor classification. The training progress mentioned above yielded the following evaluation results in the form of a confusion matrix.

Based on Figure 3 and Figure 4, the training progress mentioned above, the evaluation of the models revealed significant accurate results. Classic layer architecture achieved an accuracy of 94.55%, while the implementation of the ResNet50 architecture resulted in an improved accuracy of 99.88%. These findings indicate the superiority of the Res-Net50 architecture in accurately classifying brain tumors compared to the Classic layer architecture.

The higher accuracy obtained with ResNet50 demonstrates its effectiveness in capturing intricate patterns and features in the dataset, leading to more precise tumor classification. The training progress mentioned above yielded the following evaluation results in the form of a confusion matrix.

**Figure 5:**
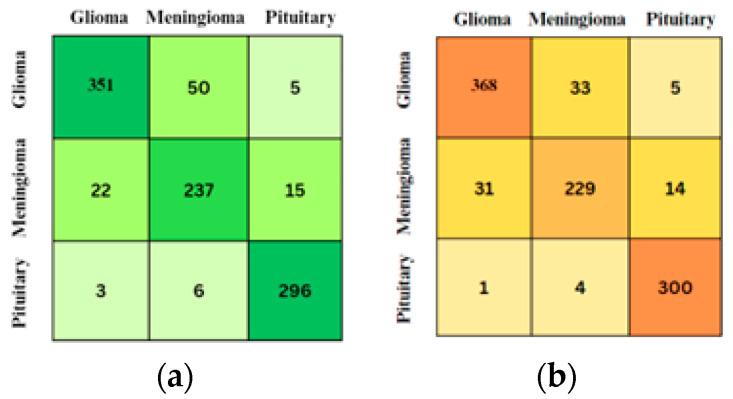
Comparison of Confusion Matrix. (**a**) Confusion matrix with Classic CNN. (**b**) Confusion matrix with Resnet-50.

**Figure 6:**
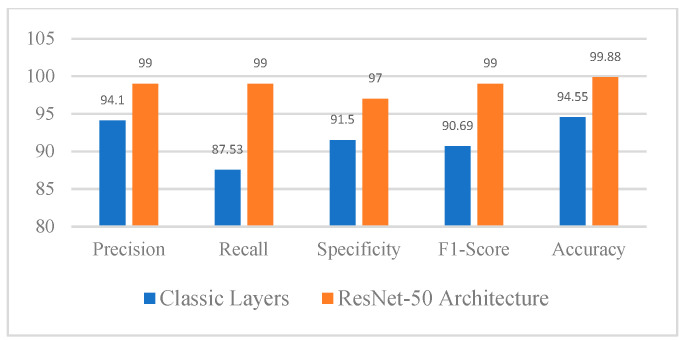
Results of CNN Model Evaluation.

Based on Figure 5 and Figure 6, utilizing Equations (4)–(8), the evaluation of the model resulted in the following metrics: Recall, Precision, Specificity, and F1-score.

**Figure 7:**
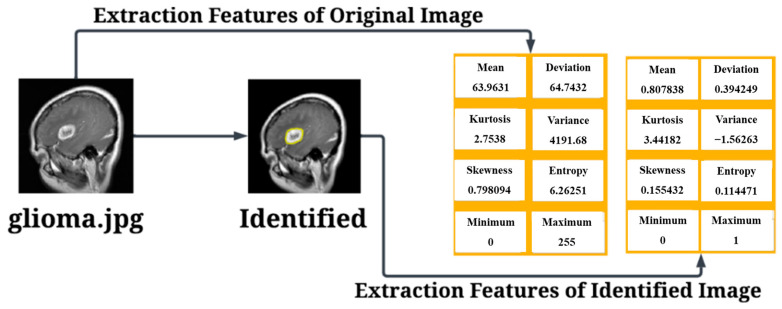
Testing Results for Glioma Tumor.

The results of Glioma Tumor can be seen below in Figure 7.

**Figure 8:**
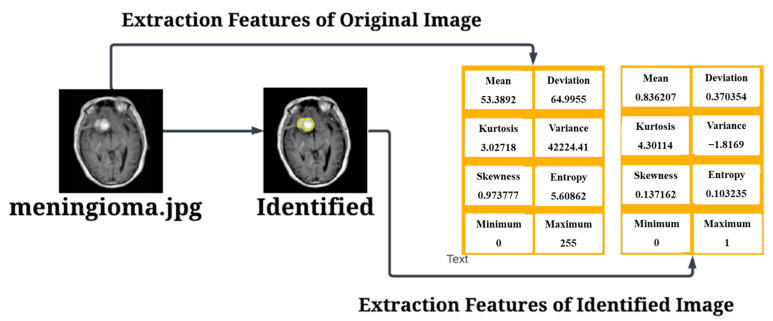
Testing Results for Meningioma Tumor.

After conducting the evaluation, the next step involved the identification of tumors and the extraction of relevant features. The results of the tumor identification process are presented in Figure 8, showcasing the successfully detected tumors and their corresponding extracted features.

**Figure 9:**
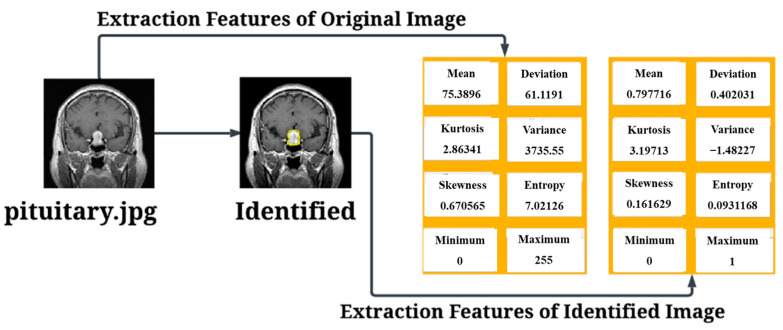
Testing Results for Pituitary Tumor.

The results of the Pituitary Tumor are presented in Figure 9. In this experiment, we conducted 6 trials using ResNet-50 architecture and classic layers, and the results showed an exceptional success rate of 100% with an overall True Positive classification.

This indicates that our model is highly effective in identifying and classifying all types of brain tumors present in the dataset.

The obtained results demonstrate a high level of reliability in the classification process and provide evidence that the ResNet-50 architecture performs exceptionally well in this task.

The final stage of this research involves the classification of three types of brain tumors from the previously described dataset, where the classification is based on the ResNet-50 architecture, and the obtained results are presented in Table 2.

Upon concluding the testing phase, a comparative analysis was conducted with other studies using the same dataset but employing different models, as depicted in Table 3. In this Research based on Table 3, the utilization of Convolutional Neural Networks (CNNs) employing the ResNet-50 architecture was investigated for classification. was meticulously acquired and subsequently partitioned into an 80% training set and a 20% testing set.

The CNN model underwent rigorous training on the designated training set, to discern intricate patterns and extract salient features inherent in the data, thereby enabling it to make accurate predictions on previously unseen samples. A comprehensive data set comprises a total of 4922 samples. The ResNet-50 architecture, renowned for its profound depth and residual network design, was deliberately selected owing to its inherent capacity to effectively capture and model complex hierarchical representations. The model’s training process encompassed iterative fine-tuning of the network’s weights and biases, with the aim of minimizing prediction errors and optimizing classification performance. Subsequently, the model was thoroughly evaluated on the reserved testing set, meticulously scrutinizing its generalization ability and overall accuracy. The empirical findings derived from this research endeavor afford profound insights into the effectiveness of CNNs, particularly when harnessed in conjunction with the ResNet-50 architecture, in classification tasks of paramount importance.

## 5. Conclusions

The utilization of the ResNet50 architecture in brain tumor classification demonstrates a significant advantage over classic CNN architecture. ResNet50 achieves an impressive accuracy of 99.88%, outperforming the classic architecture, which only attains 94.55%. Although both architectures achieved an equal number of true positives across the experiments, ResNet50 exhibits superior accuracy by minimizing the number of false negatives during testing. This highlights the benefits of employing deeper and more complex architectures, as ResNet50 enhances classification precision and reduces diagnostic errors, which could significantly impact patient treatment and prognosis. The findings contribute to the field of medical imaging by showcasing the potential of CNN-based approaches to support accurate and efficient tumor classification. These advances can facilitate early diagnosis, assist in treatment planning, and enable better patient monitoring, ultimately improving healthcare outcomes. Further efforts to optimize the performance of CNN models could focus on fine-tuning hyperparameters and integrating advanced data augmentation techniques to improve model robustness.

Several areas for future research in brain tumor classification using CNNs can be pursued. A promising direction is the integration of multi-modal imaging data, such as combining MRI with PET or CT scans, to provide a more comprehensive view of tumor characteristics and improve diagnostic precision. Investigating the application of transfer learning remains essential, as utilizing pre-trained models on large-scale datasets allows for cross-domain knowledge transfer, addressing the challenge of limited annotated data and enhancing model performance. Approaches such as visualizing activation maps, identifying key features, or generating saliency maps will increase the transparency and clinical trustworthiness of these models. Furthermore, collaborative efforts among research institutions can lead to the creation of standardized datasets and benchmarks, fostering data sharing, reproducibility, and comparative evaluations across different models. These efforts will facilitate the continuous development of accurate and reliable CNN-based solutions for brain tumor classification.

## Figures and Tables

**Table 1:** Units for Magnetic Properties.

Parameters	Value	Classic Layers	Resnet50 Layers
Optimizer	Adam	Input Image (200 × 200 × 1)	Input Image (200 × 200 × 1)
MaxEpoch	24	Convolutional (3 × 3 × 8) Batch Normalization ReLU	Convolutional Layer (7 × 7 × 64) Batch Normalization Layer ReLU
Validation frequency	30	Max Pooling	Max Pooling
InitialLearnRate	0.001	Convolutional Layer (3 × 3 × 16) Batch Normalization ReLU	Residual Block (64, 64) (64, 64) (64, 64)
MiniBatchSize	64	Max Pooling	Residual Block (128, 128) (128, 128) (128, 128)
Execution by GPU		Convolutional (3 × 3 × 32) Batch Normalization ReLU	Residual Block (256, 256) (256, 256) (256, 256)
		Max Pooling	Residual Block(512, 512) (512, 512) (512, 512)
		Fully Connected (3)	Average Pooling Layer (1 × 1)
		Softmax Layer	Fully Connected Layer (3)
		Classified	Softmax Classified

**Table 2:** Classification Results.

Image	Method	Results	TP or TN
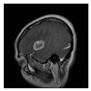	Classic Layer	Glioma	TP
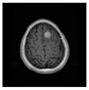	Classic Layer	Meningioma	TP
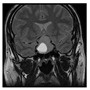	Classic Layer	Pituitary	TP
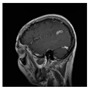	ResNet-50 Layers	Glioma	TP
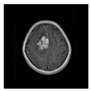	ResNet-50 Layers	Meningioma	TP
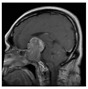	ResNet-50 Layers	Pituitary	TP

**Table 3:** Comparison with Related Research.

Ref.	Accuracy	Precision	Recall	F1-Score
[7]	97.80%	97%	97%	-
[4]	98.91%	98.28%	99.75%	99%
[22]	99.34%	99%	99%	98%
[15]	99.85%	98.16%	98.17%	98.21%
Proposed	99.88%	99%	99%	99%

## Data Availability

The utilization of the Brain tumor dataset obtained from Kaggle [24,25], which comprises MRI images with a resolution of 512 × 512 × 1, is a valuable resource for studying and analyzing brain tumors.
